# Sequence‐Based Prediction of Promiscuous Acyltransferase Activity in Hydrolases

**DOI:** 10.1002/anie.202003635

**Published:** 2020-05-11

**Authors:** Henrik Müller, Ann‐Kristin Becker, Gottfried J. Palm, Leona Berndt, Christoffel P. S. Badenhorst, Simon P. Godehard, Lukas Reisky, Michael Lammers, Uwe T. Bornscheuer

**Affiliations:** ^1^ Department of Biotechnology & Enzyme Catalysis Institute of Biochemistry University of Greifswald 17487 Greifswald Germany; ^2^ Institute of Bioinformatics University Medicine Greifswald 17487 Greifswald Germany; ^3^ Department of Synthetic and Structural Biochemistry Institute of Biochemistry University of Greifswald 17487 Greifswald Germany

**Keywords:** acylation, acyltransferases, biocatalysis, esterases, transesterification

## Abstract

Certain hydrolases preferentially catalyze acyl transfer over hydrolysis in an aqueous environment. However, the molecular and structural reasons for this phenomenon are still unclear. Herein, we provide evidence that acyltransferase activity in esterases highly correlates with the hydrophobicity of the substrate‐binding pocket. A hydrophobicity scoring system developed in this work allows accurate prediction of promiscuous acyltransferase activity solely from the amino acid sequence of the cap domain. This concept was experimentally verified by systematic investigation of several homologous esterases, leading to the discovery of five novel promiscuous acyltransferases. We also developed a simple yet versatile colorimetric assay for rapid characterization of novel acyltransferases. This study demonstrates that promiscuous acyltransferase activity is not as rare as previously thought and provides access to a vast number of novel acyltransferases with diverse substrate specificity and potential applications.

## Graphical abstract

**What's the score?** Promiscuous acyltransferase activity of esterases strongly correlates with active‐site hydrophobicity. A hydrophobicity scoring concept was developed that enables the accurate prediction of promiscuous acyltransferase activity simply from the amino acid sequence of the enzyme cap domain.

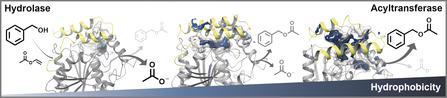

## Introduction

**Figure 1:**
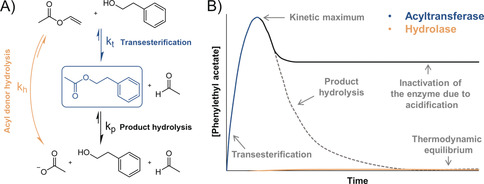
A) Enzymatic acetylation of 2‐phenylethanol using vinyl acetate as an acyl donor. Acyl‐donor hydrolysis (*k*_h_) and acyl transfer (*k*_t_) are competing reactions. B) Schematic progression of product concentration in kinetically controlled reactions catalyzed by promiscuous acyltransferases and conventional hydrolases. The dotted line represents the product hydrolysis that would be observed if the enzyme were not inactivated by acidification.

Due to their robustness, lack of cofactor requirements, and catalytic promiscuity, hydrolases have gained great popularity in the field of biocatalysis.^1^ In aqueous solution, water usually acts as the nucleophile, thereby resulting in the hydrolysis of esters or lipids (fats and oils). The ability of some hydrolases to also maintain activity in a water‐free organic environment has enabled a broad spectrum of synthetically interesting transformations like (*trans*‐)esterification and amide formation.^2^ However, some hydrolases can catalyze acyl transfer even in the presence of bulk water, thereby providing a greener alternative by obviating the need for expensive and hard‐to‐dispose‐of organic solvents. For example, penicillin acylases have been used for decades in a kinetically controlled reaction to synthesize β‐lactam antibiotics in water.^3^, ^4^ More recently, lactam synthesis from ω‐amino fatty acids in water was described.^5^ Apart from that, the ability to catalyze acyl transfer in water has only been demonstrated for a few lipases related to *Pseudozyma antarctica* lipase A (CAL‐A), including the widely studied CpLIP2 from *Candida parapsilosis*, and an aryl esterase from *Mycobacterium smegmatis* (MsAcT).^6^, ^7^ For example, CAL‐A can be used for the production of fatty acid ethyl esters from plant oil in an aqueous environment.^8^ Because of their acyltransferase activity in water, these enzymes can also be used in cascade reactions with other enzymes. Thus, the acyltransferase activity of CAL‐A has been used by us to enhance the production of *ϵ*‐caprolactone oligomers from cyclohexanol. Here, CAL‐A catalyzes the oligomerization of *ϵ*‐caprolactone, thereby reducing product inhibition and shifting the equilibrium towards product formation.^9^ By following the same strategy, MsAcT was used in a cascade reaction with a transaminase to synthesize *N*‐benzylacetamide from benzaldehyde.^10^ MsAcT expands the range of possible reactions by catalyzing transesterification from small and low‐priced acyl donors like ethyl acetate towards a broad range of organic nucleophiles at a much higher rate than hydrolysis. Figure 1 A shows the biocatalytic acetylation of 2‐phenylethanol, a typical reaction that can be catalyzed by a promiscuous acyltransferase like MsAcT. The use of vinyl esters as acyl donors makes the reaction virtually irreversible. Figure 1 B shows the schematic progression of product concentration in this reaction for promiscuous acyltransferases in contrast to conventional hydrolases. The acylated product is only transiently accumulated, and the maximum achievable conversion is determined by the ratio of the transfer to hydrolysis rates (*k*_t_/*k*_h_) and the rate of product hydrolysis (*k*_p_).^11^ Hydrolysis is thermodynamically favored and leads to progressive acidification of the reaction, which can eventually result in the inactivation of the enzyme.^12^ The product is then not enzymatically hydrolyzed anymore, and its concentration remains constant.

Unlike naturally occurring CoA‐dependent acyltransferases, which often show virtually no hydrolase activity, promiscuous acyltransferases can hydrolyze both the acyl donor and the transesterification product. Therefore, the product of transesterification only transiently accumulates, and high conversions can only be achieved in a kinetically controlled reaction (Figure 1 B). Conventional hydrolases do not show a transient kinetic maximum above the thermodynamic equilibrium under the same conditions.^13^ To judge the efficiency of promiscuous acyltransferases in a specific kinetically controlled reaction, the acyl transfer to hydrolysis ratio (*k*_t_/*k*_h_; Figure 1 A) has become a commonly accepted parameter.^3^, ^14^

The few known promiscuous acyltransferases come with several synthetic limitations. Even though some CAL‐A‐related acyltransferases show great efficiency, their application is limited to long‐chain fatty acid esters as acyl donors, with CpLIP2 even being inhibited by short‐chain esters like ethyl acetate.^15^ MsAcT is generally not enantioselective and does not accept more polar substrates like amino acids.^10^, ^12^, ^16^

Due to the small number of known acyltransferases, the structural prerequisites for efficient acyltransferase activity in water are also not fully understood. Jiang et al. discussed the role of the immediate surrounding of the catalytic triad and proposed a mechanism for acyltransferases that involves the inactivation of attacking water molecules by acidic backbone amide hydrogens.^17^ For MsAcT, it has been speculated that the highly hydrophobic microenvironment formed by its oligomeric structure favors the binding of organic nucleophiles over water.^7^, ^18^ Supporting this, studies of CAL‐A and CpLIP2 revealed that substitution of active site residues by more hydrophobic residues improves acyltransferase activity.^8^, ^19^

We recently reported a high‐throughput assay for identifying more enzymes with acyltransferase activity in water.^20^ By screening several hydrolases using this assay, we identified a member of the family of bacterial hormone‐sensitive lipases (bHSLs), Est8, as a promiscuous acyltransferase.^20^, ^21^ Based on a detailed structure–function analysis of Est8^22^ and several homologues, the present study aims to clarify molecular prerequisites for promiscuous acyltransferase activity, thereby enabling sequence‐based prediction of this remarkable phenomenon.

## Results and Discussion

**Figure 2:**
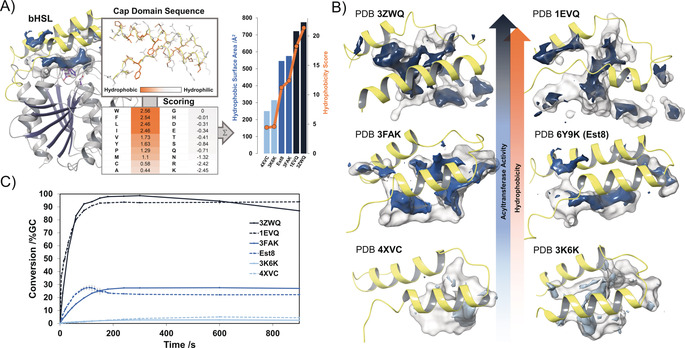
A) An overview of our hydrophobicity scoring system, using the structure of Est8 (PDB ID: 6Y9K) as an example, with its catalytic triad highlighted in purple. The 45 N‐terminal residues of bHSL sequences were used to calculate hydrophobicity scores by calculating the sum of values taken from the hydrophobicity scale of Abraham & Leo.^23^ The hydrophobicity scores (4XVC=4.4, 3K6K=4.6, Est8=11.2, 3FAK=12.4, 1EVQ=18.2, 3ZWQ=21.4) correlate well with the hydrophobic surface areas calculated from crystal structures (correlation coefficient *r*=0.98). B) Visualization of hydrophobicity inside the substrate‐binding pockets of Est8 and its homologues. Darker shades of blue indicate increasing acyltransferase activity and hydrophobicity. Hydrophobic surface areas are colored accordingly. C) Enzymatic acetylation of 2‐phenylethanol (20 mm) using a ten‐fold excess of vinyl acetate (200 mm) as an acyl donor. Reactions were carried out in 200 mm buffer at the enzymes’ pH optima.

Est8 is the first member of the family of bacterial hormone‐sensitive lipases (bHSLs) for which promiscuous acyltransferase activity in water has been reported. In order to elucidate the structural basis for this activity, we solved the crystal structure of Est8 (PDB ID: 6Y9K; Table S1 in the Supporting Information). We observed that its substrate‐binding pocket consists of a well‐defined tunnel, which leads from the protein surface to the active site harboring a catalytic triad (Ser^146^, Glu^240^, His^270^, Figure 2 A). Remarkably, this tunnel is highly hydrophobic due to the presence of several methionine residues that are part of the N‐terminal cap domain, a helix‐turn‐helix motif covering the active site. As discussed for CpLIP2, CAL‐A, and MsAcT, we assumed that active‐site hydrophobicity also plays a role for the acyltransferase activity of Est8. As typical for bHSLs, the cap domain forms the most significant part of the substrate‐binding pocket of Est8. In contrast to the conserved C‐terminal catalytic domain, the cap domain is highly variable (Figure S1 in the Supporting Information). Because of this structural feature, we postulated that active‐site hydrophobicity of bHSLs could be estimated from the amino acid sequence of the cap domain. For that purpose, we developed a sequence‐based hydrophobicity scoring system for bHSLs (Figure 2 A) based on a hydrophobicity scale for amino acids.^23^ On this scale, hydrophobic residues have positive values, while polar and charged residues have negative values and thus contribute as penalties. We summed the values for the 45 N‐terminal residues, which form the cap domain, as visualized in Figure 2 A. Hydrophobic pockets are thus expected to have high scores while hydrophilic pockets have low or even negative scores. In order to demonstrate the ability of the hydrophobicity score to reflect actual active site hydrophobicity, we analyzed the crystal structures of five other bHSLs (PDB IDs: 3ZWQ, 1EVQ, 3FAK, 4XVC, and 3K6K). Hydrophobic pocket surface areas (visualized in Figure 2 B) calculated from the crystal structures were plotted against the hydrophobicity scores, revealing nearly perfect coherence (correlation coefficient *r*=0.98, Figure 2 A).

The same set of bHSLs was experimentally analyzed for promiscuous acyltransferase activity in a model reaction (Figure 1 A) involving the acetylation of 2‐phenylethanol in the presence of a ten‐fold excess of vinyl acetate as acyl donor (Figure 2 C). Formation of the product, 2‐phenylethyl acetate, was quantified by gas chromatography (GC). For Est8, product hydrolysis became more dominant than acyl transfer quickly after reaching a kinetic maximum at low concentrations (ca. 27 % conversion) of 2‐phenylethyl acetate. On the way to reaching its thermodynamic equilibrium, the reaction is rapidly quenched by acidification, thus indicating high hydrolase activity. Enzymes with hydrophobicity scores lower than Est8, such as 3K6K^24^ and 4XVC,^25^ behaved as conventional hydrolases with nearly no acyltransferase activity (<5 % conversion) in water. 3FAK,^26^ an esterase with a hydrophobicity score equal to that of Est8, showed acyltransferase activity comparable to Est8 (ca. 27 % conversion). Most strikingly, enzymes with hydrophobicity scores higher than that of Est8, specifically 1EVQ^27^ and 3ZWQ,^28^ were excellent acyltransferases that could nearly fully convert 2‐phenylethanol into 2‐phenylethyl acetate (Figure 2 C).

Even though the prediction of promiscuous activities is commonly considered to be extremely difficult,^29^ the active‐site hydrophobicity calculated from crystal structures seems to be highly predictive of acyltransferase activity in water. Consequently, the hydrophobicity score, which accurately reflects active‐site hydrophobicity, can be used to predict promiscuous acyltransferase activity within this enzyme family.

**Figure 3:**
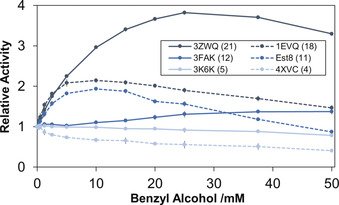
Changes in relative activity of bHSLs in the presence of different concentrations of benzyl alcohol as an acyl acceptor in the *p*NPA‐acyltransferase assay. Rounded hydrophobicity scores are given in parentheses. The ranking of acyltransferase activities agrees with that determined using GC analysis (Figure 2 C).

In order to gain more insight into the acyltransferase efficiency of the newly identified acyltransferases, we needed a more sophisticated assay that provides information about the relative rates of acyl transfer to acyl‐donor hydrolysis. However, our oligocarbonate assay^20^ provides only qualitative information. Mestrom et al. recently reported a coupled spectrophotometric acyltransferase assay, which we could not use since the employed alcohol dehydrogenase precipitates in the presence of organic nucleophiles other than benzyl alcohol. Therefore, we developed a robust and versatile acyltransferase assay using *para*‐nitrophenyl esters as acyl donors. Our assay has the advantage of being compatible with virtually any organic nucleophile as the acyl acceptor. Upon formation of the acyl‐enzyme intermediate, *para*‐nitrophenolate (*p*NP) is released and can be quantified by measuring the increase in absorbance at 405 nm. In the absence of any other nucleophile than water, the half‐life of the acyl‐enzyme intermediate depends only on the rate of hydrolysis. However, if an organic nucleophile is preferred over water, the half‐life of the acyl‐enzyme intermediate is expected to be shorter in its presence, resulting in an overall faster release of *p*NP (Figure S3A). To show that the increased release of *p*NP is directly correlated with enzymatic transesterification, and not due to a nonspecific acceleration of *p*NPA hydrolysis, the formation of the transesterification product, benzyl acetate, was confirmed by GC analysis. A direct correlation between the rate of *p*NP release at different benzyl alcohol concentrations and benzyl acetate formation was observed (Figure S3B). The above‐described reactions were carried out for Est8 and its homologues using both benzyl alcohol (Figure 3) and 2‐phenylethanol (Figure S7) as acceptors, and the increase in activity relative to hydrolysis was plotted as a function of the organic nucleophile concentration.

The fact that the ranking of acyltransferase activities is the same with both acyl acceptors (Figure 3 and Figure S7) demonstrates the suitability of the colorimetric assay for determining whether a hydrolase has acyltransferase activity or not. Moreover, we observed that the choice of alcohol can have a tremendous influence on acyltransferase activity, which is natural for an enzyme‐catalyzed reaction. In general, higher relative activities were observed when using benzyl alcohol (Figure 3) instead of 2‐phenylethanol (Figure S7).

For example, 3ZWQ showed three‐fold higher acyltransferase activity with benzyl alcohol, outperforming 1EVQ. In congruence with the time curves shown in Figure 2 C, the two enzymes show comparable activity in the colorimetric assay when using 2‐phenylethanol as acyl acceptor.

**Figure 4:**
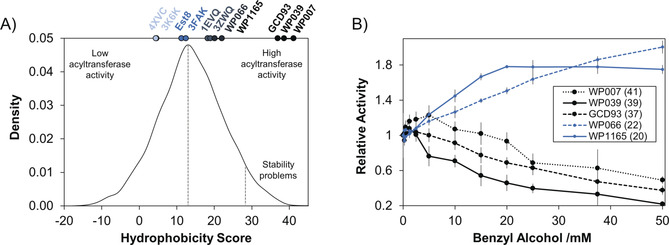
A) Density plot showing the relative frequency of hydrophobicity scores within the sequence library obtained from a BLAST search based on the amino acid sequence of 3FAK. The values range from −25 to 45, and the mean score is 13.65. The positions of Est8 and its homologues on the distribution are indicated. B) Relative activities are plotted as a function of benzyl alcohol concentration for several previously uncharacterized bHSLs. Rounded hydrophobicity scores are given in parentheses for each enzyme. Consistent with the prediction, WP066 and WP1165 (blue), which have hydrophobicity scores similar to 1EVQ and 3ZWQ, show good acyltransferase activity while the overall activities of those with scores in the upper quartile are negatively affected by the presence of higher concentrations of the alcohols.

In order to put the hydrophobicity scores of these enzymes in a broader context, we generated a sequence library comprising 20 000 sequences by performing a BLAST search with 3FAK as the query sequence. The library was filtered by sequence length, redundant entries were removed, and hydrophobicity scores were calculated for the remaining 6500 sequences. The calculated scores range from −22 to 45, with an average of 13.65 (Figure 4 A).

Since bHSLs with scores (see Figure 2 A) lower than the mean value showed low (Est8 and 3FAK) or virtually no (4XVC and 3K6K) acyltransferase activity, it seemed unlikely that good acyltransferases have scores below the average. In contrast, bHSLs with extremely high scores have high numbers of hydrophobic residues in the cap domain. Therefore, they are likely to be unstable in solution since it is known that the rate of aggregation of proteins increases with exposed hydrophobic surface area.^30^ Consequently, we propose that good acyltransferases within the family of bHSLs have hydrophobicity scores above average but below the upper quartile.

Following the predicted activity of database homologues, a selection of proteins (WP1165, WP066, GCD93, WP039, WP007) with above‐average scores was expressed, purified, and investigated using the colorimetric acyltransferase assay. Supporting the reliability of the method, WP1165, and WP066, which have scores similar to 1EVQ and 3ZWQ, also have similar acyltransferase activities (Figure 4). Interestingly, WP066 had good activity with benzyl alcohol (Figure 4) but did not accept 2‐phenylethanol as substrate (Figure S7). Remarkably, activity is further elevated for WP066 with benzyl alcohol concentrations exceeding 40 mm. For proteins with a very hydrophobic cap domain (GCD93, WP039, and WP007), we indeed observed major problems with protein expression and stability. For most of them, the relative activity quickly dropped already at low alcohol concentrations, thus suggesting low stability. While Est8 and the homologues described above maintain their activity for over a month, purified WP007 precipitates within minutes after purification, resulting in a complete loss of activity.

Nevertheless, lyophilized crude lysate of WP007 was shown to have significant acyltransferase activity, acetylating more than 95 % of 20 mm 2‐phenylethanol (Figure S8). Thus, prediction of acyltransferase activity using the hydrophobicity scoring system turned out to be highly accurate, and the newly discovered enzymes, notably identified by testing less than a dozen candidates, represent synthetically useful biocatalysts.

## Conclusion

The results of this study provide evidence that promotion of promiscuous acyltransferase activity by active‐site hydrophobicity is a general concept for hydrolases. We demonstrated that active‐site hydrophobicity can be estimated from the amino acid sequence of the N‐terminal cap domain for bHSLs. By using our hydrophobicity scoring, we were able to identify five bHSLs with much higher acyltransferase activity than Est8. This suggests that, following the prediction, at least 2000 other promiscuous acyltransferases await discovery in the sequence library created. We have also introduced a robust and versatile colorimetric acyltransferase assay for the rapid identification and characterization of acyltransferase activity in esterases.

## Supporting information

As a service to our authors and readers, this journal provides supporting information supplied by the authors. Such materials are peer reviewed and may be re‐organized for online delivery, but are not copy‐edited or typeset. Technical support issues arising from supporting information (other than missing files) should be addressed to the authors.

Supplementary
