# Supplementary material for: Sequence‐Based Prediction of Promiscuous Acyltransferase Activity in Hydrolases
Source: Angew Chem Int Ed Engl. 2020 May 11;59(28):11607–12. doi: 10.1002/anie.202003635 (PMC7383625; doi:10.1002/anie.202003635)
Supplement: Supplementary file 1 — Supplementary [file ANIE-59-11607-s001.pdf]

Angewandte Chemie
Eine Zeitschrift der Gesellschaft Deutscher Chemiker

Supporting Information

Sequence-Based Prediction of Promiscuous Acyltransferase Activity in Hydrolases

*Henrik Müller, Ann-Kristin Becker, Gottfried J. Palm, Leona Berndt, Christoffel P. S. Badenhorst, Simon P. Godehard, Lukas Reisky, Michael Lammers, and Uwe T. Bornscheuer**

anie_202003635_sm_miscellaneous_information.pdf

Table of Contents

Experimental Procedures 3
Materials 3
Expression and Purification of His_6-tagged bHSLs 3
SDS-PAGE Analysis 3
Crystallization of Est8 and Data Collection 3
Structure Solution and Refinement 4
In Silico Prescreening 5
In silico Analysis of the Substrate Binding Pockets 5
Biocatalysis reactions and GC Analysis 5
Colorimetric Acyltransferase Assay 5
Additional Results 6
Sequences 10
References 13
Author Contributions 14

Experimental Procedures

Materials

Benzyl alcohol and 2-phenylethanol were purchased from FLUKA (Buchs, Switzerland) and vinyl acetate from ACROS ORGANICS (Geel, Belgium). All other chemicals and solvents were purchased from Sigma, VWR, or Carl Roth and were used without further purification. Synthetic genes were codon-optimized for expression in *Escherichia coli* and subcloned into pET-28a(+) by BioCat GmbH (Heidelberg, Germany). Est8 was obtained from BRAIN AG (Zwingenberg, Germany) and was expressed from pET-26 and PestE from pET-21, respectively. All plasmids encoded C-terminal His_6-tags.

Expression and Purification of His_6-tagged bHSLs

Chemically competent *E. coli* BL21(DE3) cells were transformed with expression vectors and plated on LB agar containing 50 μg/mL kanamycin. Pre-cultures (5 mL of LB containing 50 μg/mL kanamycin) were inoculated with single colonies and incubated overnight (37°C, 180 rpm). TB medium (200 mL containing 50 μg/mL kanamycin) was inoculated with 0.2% (v/v) of the pre-culture. The cultures were incubated (37°C, 180 rpm) until they reached an OD_{600} of 0.6. Protein expression was induced by addition of isopropyl-β-D-thiogalactoside to a final concentration of 0.4 mM, followed by incubation at 20°C (180 rpm) for 16 h. Cells were harvested by centrifugation at 4000 *g* and 4°C for 30 min and washed with 30 mL of 50 mM potassium phosphate (pH 7.4). Washed cell pellets were stored at -20°C for later use.

Cell pellets were resuspended in ~20 mL lysis buffer (50 mM potassium phosphate, 300 mM sodium chloride, pH 8.0) and lysed by sonification on ice (5 cycles of 1 min sonication (40% intensity, 50% pulsed cycle) followed by 1 min incubation on ice) using a SONOPULS HD 2070 (BANDELIN electronic GmbH & Co. KG, Berlin, Germany). Lysates were clarified by centrifugation (10000 *g*, 4°C, 30 min). The His_6-tagged proteins were purified by immobilised metal-affinity chromatography using 3 ml of Roti®garose-His/Ni Beads (Carl Roth, Karlsruhe, Germany). The Ni-NTA resin was washed with deionized water and equilibrated with lysis buffer. The lysates were applied by gravity and the flow-through discarded. Unspecifically bound proteins were removed by excessive washing of the resin with washing buffer (50 mM potassium phosphate, 300 mM sodium chloride, 20 mM imidazole, pH 8.0). The recombinant proteins were eluted with approximately 6 ml of elution buffer (50 mM potassium phosphate, 300 mM sodium chloride, 300 mM imidazole, pH 8.0). Elution fractions were stored at 4°C after being desalted using PD-10 desalting columns (GE Healthcare, UK) equilibrated with storage buffer (200 mM potassium phosphate, pH 8.0). For crystallization experiments, Est8 was further purified by gel filtration on a Superdex200 16/60 column (GE Healthcare, Freiburg, Germany) with storage buffer. Protein concentrations were determined by measuring absorbance at 280 nm using a NanoDrop™ (Thermo Fisher, Germany) device. Theoretical extinction coefficients and molecular weights were calculated from the protein sequences using ExPASy ProtParam.[1] If necessary, proteins were further concentrated using Vivaspin™ concentrators with 10 kDa cut-off (Sartorius, Germany).

SDS-PAGE Analysis

The purity of protein samples was analysed by SDS-PAGE. Samples of the purified proteins were denatured by heating (95°C, 10 min) in Laemmli-buffer[2] followed by centrifugation (20800 *g*, 5 min). The proteins were separated on 12.5% acrylamide gels at a constant voltage of 120 V. The gels were stained using Coomassie Brilliant Blue G-250.

Crystallization of Est8 and Data Collection

Est8 in phosphate buffer (200 mM, pH 8.0) was concentrated to 30 mg/mL and screened for crystallization conditions by mixing 0.3 μL protein with 0.3 μL well solution from JBScreen Classic Kits 1-10. The successful condition (25% PEG 4000, 0.1 M sodium citrate, pH 5.6, and 0.2 M ammonium sulfate) was refined in larger hanging drops (2 μL protein + 2 μL reservoir solution). A crystal from 29% PEG 4000, 0.1 M ammonium sulfate, and 0.1 M sodium citrate, pH 5.6, was cryo-cooled in liquid nitrogen and measured at BESSY beamline 14.2 at 100 K. Data were processed using XDSAPP.[3]

Structure Solution and Refinement

Molecular replacement was done with monomer A of PDB entry 4XVC using Phaser.[4] The resulting solution for 4 monomers in the asymmetric unit had an LLG of 3391 and an R_{cryst} of 0.442. Refinement was performed with *REFMAC5* 5.8.0258. Residues 1-43 had to be manually remodeled. TLS refinement did not improve the geometry and was omitted.

Table 1: Statistics of the X-ray diffraction data collection and structure refinement.

**Data collection**	
Detector	PILATUS3 S 2M
Radiation source	BESSY, Beamline 14.2
Wavelength	0.9184 Å
Resolution range (last shell)	49.5 – 2.30 Å (2.43-2.30 Å)
Space group / *a*-axis / *b*-axis / *c*-axis / β	$P2_1$ / 78.2 Å / 78.2 Å / 112.6 Å / 100.7°
Number of independent reflections	59126 (9359)
Completeness (last shell)	98.9 % (97.8 %)
Redundancy	7.0 (7.1)
I/σ(I) (last shell)	7.8 (1.4)
Wilson B-factor	44 $Å^2$
R_{sym} (last shell)	0.194 (1.291)
R_{meas}[5] (last shell)	0.210 (1.391)
R_{pim}[6] (last shell)	0.073 (0.484)
$CC_{(1/2)}$[7] (last shell)	0.994 (0.591)

**Refinement**	
R_{cryst} / R_{free} (test data set with 2.5 % of all data)	0.2051 / 0.2389
Number of atoms of Est8 / water	8837 / 179
**Average isotropic B-factors**	
Protein main chain / side chain	44.2 / 48.0 $Å^2$
water	40.1 $Å^2$
**R.m.s. deviations from ideal geometry**	
Bond lengths	0.008 Å
Bond angles	1.63 °
Torsion angles	7.28 °
Molprobity score/percentile	2.12 / 85
Protein data bank entry	6Y9K

In Silico Prescreening

A sequence library containing more than 20,000 sequences was generated by a BLAST search, using the sequence of 3FAK as query. The sequence library was post-processed using R Studio. Sequences longer or shorter than 300 ± 10 residues were removed from the library. Redundant entries were removed, and the remaining 6,500 sequences were ranked by cap domain hydrophobicity. Hydrophobicity scores were calculated by summing the values from the hydrophobicity scale (reported by Abraham and Leo[8]) for the 45 N-terminal residues.

In silico Analysis of the Substrate Binding Pockets

The substrate-binding sites in the crystal structures of 4XVC, 3K6K, 3FAK, Est8, 3ZWQ and 1EVQ were identified and analysed using the SiteMap tool[9] of the Schrödinger Maestro software suite (Schrödinger, New York City, USA). In order to guarantee comparability, all structures were processed with the same parameters. A minimum of 15 site points per reported site were set with a total number of 5 site-point groupings using the more restrictive definition of hydrophobicity and the standard grid. Maps were always cropped 4 Å from the nearest site point. In all cases, the actual binding pocket was represented as the top hit. The hydrophobic surface areas of the top-ranked binding site were calculated from that with the isovalue set to -0.4 for all structures.

Biocatalysis reactions and GC Analysis

Purified enzymes (0.05 mg/mL final concentration), 20 mM 2-phenylethanol, and 200 mM vinyl acetate in 1 mL buffer (200 mM potassium phosphate) were mixed to yield a monophasic reaction mixture. Reactions were carried out at the pH optima of the enzymes (4XVC[10], 3K6K[11], 3FAK[12], 1EVQ[13], 3ZWQ[14]) Reactions with 3ZWQ, Est8 and 4XVC were carried out in 200 mM potassium phosphate (pH 8.0). Reactions with 3K6K were carried out in 200 mM potassium phosphate (pH 7.0). Reactions with 3FAK and 1EVQ were carried out in 200 mM CHES (pH 9.0). Reaction mixtures were shaken (1400 rpm) at room temperature (25°C). Time samples were taken after 0, 10, 20, 30, 45, 60, 90, 120, 150, 180, 240, 300, 600, and 900 s. Additional samples were taken for Est8 at 40, 50, 70, 80, 100, 110, 130, 140, 160, 170, and 200 s. To obtain reproducible results, more vigorous stirring of Est8 reactions was necessary between 0 and 150 s. At each time point, a 50 µL sample of the reaction was quenched by extraction with 500 µL methyl *tert*-butyl ether (MTBE) containing 0.5 mM acetophenone as internal standard. The mixtures were rapidly vortexed, followed by phase separation by centrifugation (15000 *g*, 1 min). Organic phases were transferred to GC vials and analysed by GC. Samples (2 µl, split ratio 29) were analysed using a GC-QP2010 SE device (Shimadzu, Duisburg, Germany) equipped with a ZB-5MSi column (30m × 0.25 mm, thickness 0.25 µm). Injector temperature was 250°C and a flow rate of 1.08 mL/min was used. Column temperature was initially at 80°C for 2 min, then increased at 8°C/min until 150°C and finally increased to 330°C at 15°C/min.

Colorimetric Acyltransferase Assay

Activity assays were carried out at 25°C in transparent 96-well polystyrene plates. Changes in absorbance at 405 nm were measured using a Tecan Plate Reader (Tecan, Männedorf, SWITZERLAND). Reactions were started by addition of 100 µL of the enzyme solution (in 200 mM potassium phosphate, pH 7.0) to 100 µl of a 2x master mix containing all other components in 200 mM potassium phosphate (pH 7.0). The final reactions contained *p*NPA (1 mM from a 2 M stock in DMSO, 0.05% DMSO in the final reactions) and 2-phenylethanol or benzyl alcohol (0, 0.25, 0.5, 1.25, 2.5, 5, 10, 15, 20, 25, 37.5, or 50 mM). Reactions were measured in triplicate and corrected by subtraction of chemical background hydrolysis of *p*NPA in the buffer (background was measured for each concentration of alcohol used). The initial slope was determined as shown in Fig. S4 and the amount of *p*NPA formed was calculated from an external calibration curve of *p*-nitrophenol (in 200 mM potassium phosphate, pH 7.0) in the range of 0.0 to 1.0 mM (Fig. S6). The acyl transfer to hydrolysis rate ratios were then calculated by dividing the specific activities obtained for the reactions with varying amounts of alcohol (acyl transfer + hydrolysis) by that obtained for the reaction without alcohol (hydrolysis). The *p*NPA acyltransferase assay has the advantage of being compatible with virtually any alcohol as acyl acceptor substrate, compared to the alcohol dehydrogenase assay recently published by Mestrom *et al.*[15] We could not use this assay because the alcohol dehydrogenase precipitates in the presence of 2-phenylethanol.

Additional Results

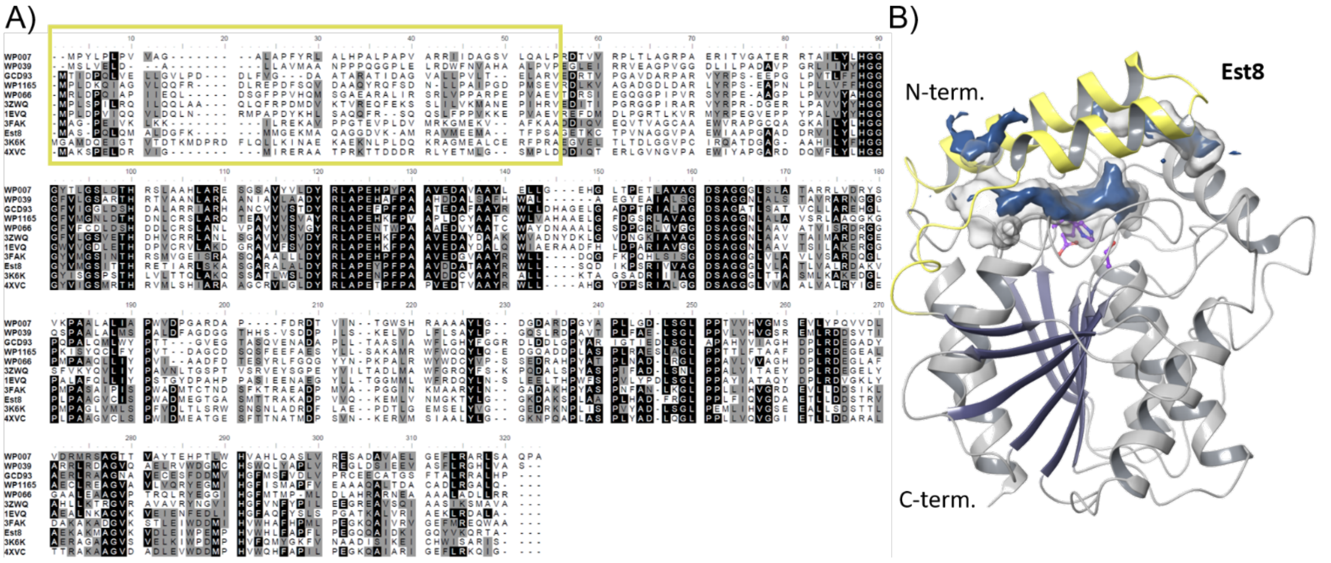

Figure S1: A) Alignment of the sequences of Est8 and homologous bHSLs. The variable cap domain sequence is highlighted by a yellow rectangle. Residues highlighted in black are identical while grey shading indicates similarity. B) The crystal structure of Est8, showing the position of the cap domain (yellow) relative to the catalytic triad consisting of Ser^{146}, Glu^{240} and His^{270} (purple carbons). Hydrophobic surface area within the substrate binding pocket is highlighted in blue.

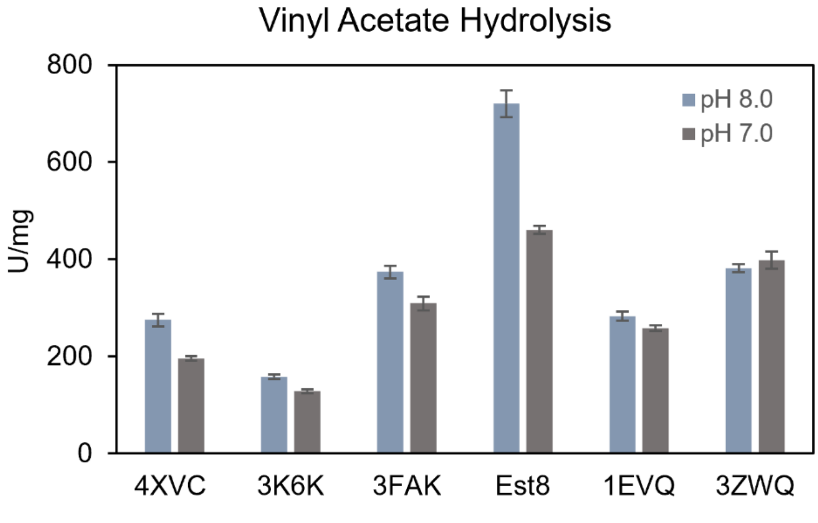

Figure S2: Specific activities for Est8 and homologs in the hydrolysis of vinyl acetate determined using the coupled spectrophotometric assay as described by Mestrom *et al.*[15] Reactions were performed in 200 mM phosphate buffer and vinyl acetate concentration was 200 mM.

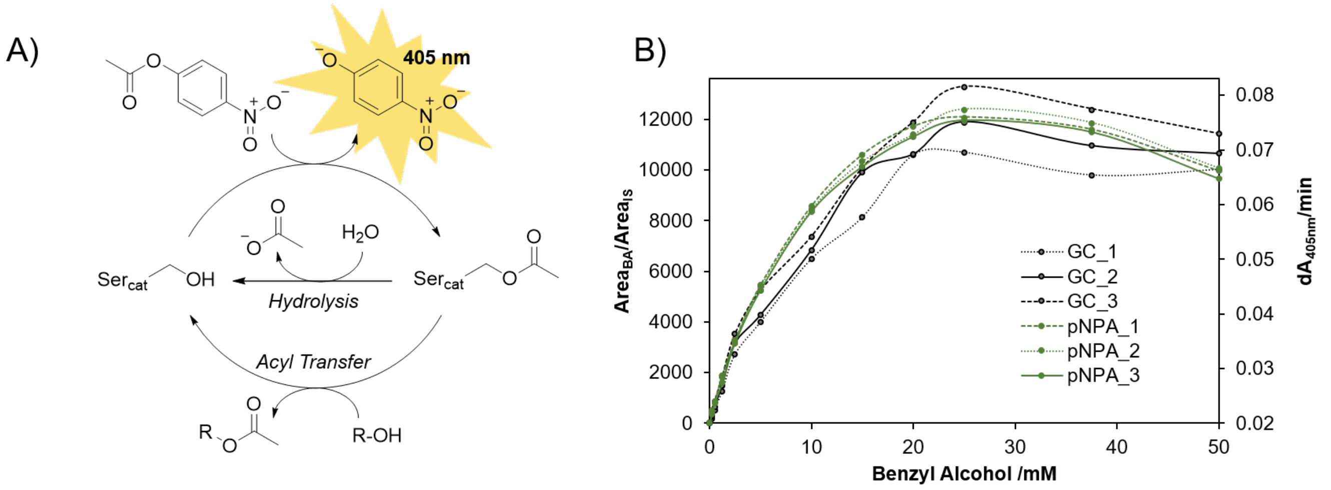

Figure S3: A) Reaction scheme of the *p*NP-acyltransferase assay. An acetyl group is transferred from *p*NPA to the enzyme via nucleophilic attack of the catalytic serine on *p*PNA, leading to the release of *p*-nitrophenolate which can be monitored at 405 nm. The acyl-enzyme intermediate formed can be attacked by either a water molecule (hydrolase activity) or an organic nucleophile like an alcohol (acyltransferase activity). A good acyltransferase would preferentially utilize the organic nucleophile over water, resulting in accelerated release of *p*-nitrophenolate in the presence of the alcohol compared to the reaction in its absence. B) GC was used to prove that the *p*NPA assay detects actual formation of benzyl acetate rather than nonspecific acceleration of *p*NPA hydrolysis. The increase in rate of 4-nitrophenolate release with increasing concentration of benzyl alcohol correlates well with the amount of benzyl acetate detected by GC.

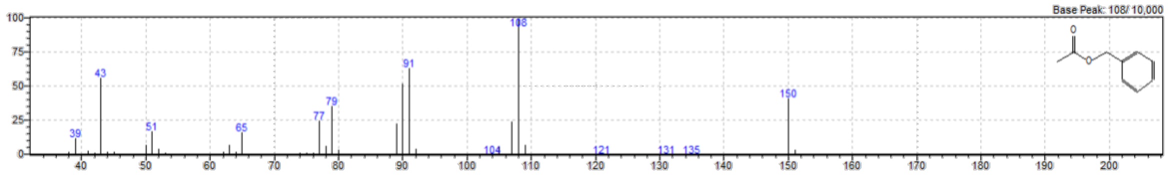

Figure S4: MS measurement after separation via GC of the reaction shown in Fig. S3 to prove that benzyl acetate is formed as product of enzyme-catalysed transesterification in the *p*NPA-acyltransferase assay. The exact mass of benzyl alcohol is 150.18 g/mol.

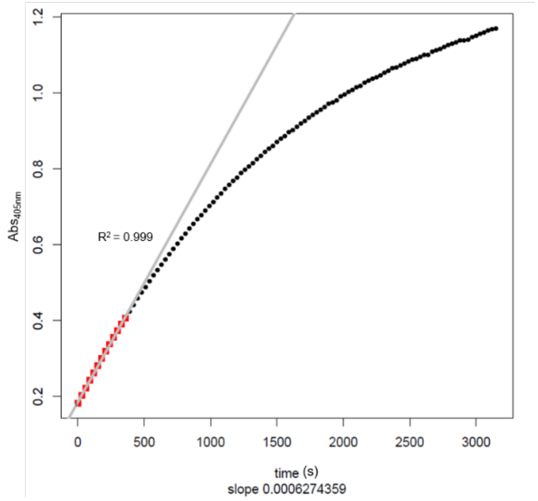

Figure S5: Slope determination in the *p*NPA-assay. An example for the reaction of 3ZWQ in the presence of 5 mM benzyl alcohol is shown. The linear slopes were determined so that R^2 in each case is ≥0.99.

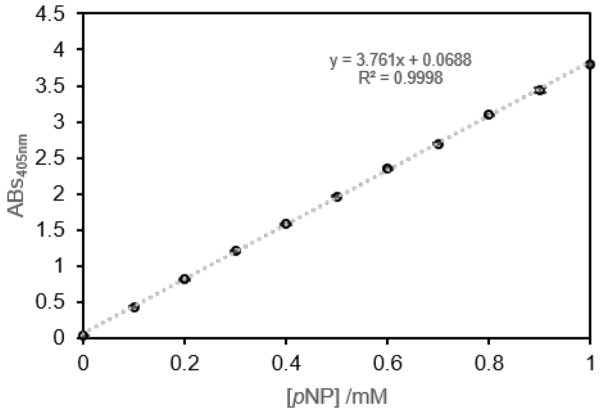

Figure S6: *p*NP calibration curve for the quantification of *p*NP by measuring the absorbance at 405 nm at different concentrations of *p*NP.

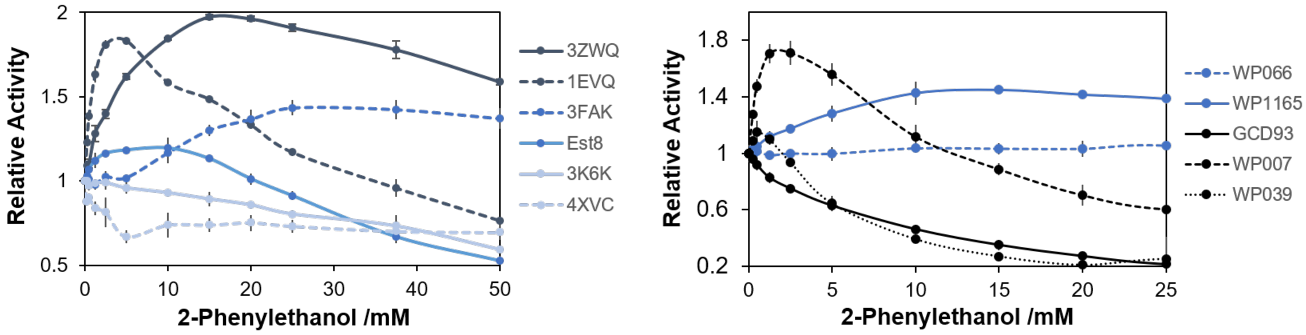

Figure S7. Changes in relative activity of bHSLs in the presence of different concentrations of 2-phenylethanol.

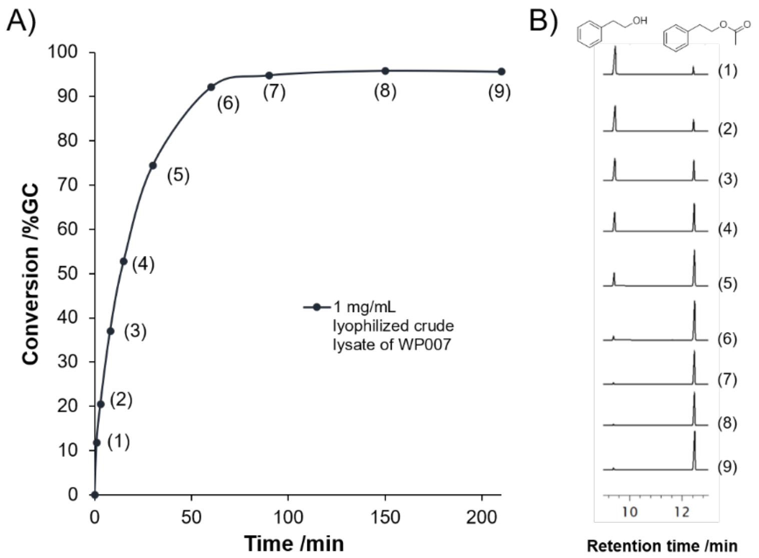

Figure S8. A) Reaction progression of the acetylation of 2-phenylethanol (20 mM) using a ten-fold excess of vinyl acetate (200 mM) as acyl donor in the presence of 1 mg/mL lyophilized crude lysate of WP007. Reactions were carried out in 200 mM potassium phosphate (pH 8.2). WP007 is able to almost fully convert 2-phenylethanol to 2-phenylethyl acetate. B) GC-chromatograms showing the conversion of the substrate to the product over time.

W	2.56	G	0
F	2.54	H	-0.01
L	2.46	D	-0.31
I	2.46	E	-0.34
V	1.73	T	-0.41
Y	1.63	S	-0.84
P	1.29	Q	-0.71
M	1.1	N	-1.32
C	0.58	R	-2.42
A	0.44	K	-2.45

Figure S9: Hydrophobicity scale introduced by Abraham & Leo.[8] Residues which are more hydrophobic relative to glycine (0) have a positive value, while residues more hydrophilic than glycine were assigned negative values.

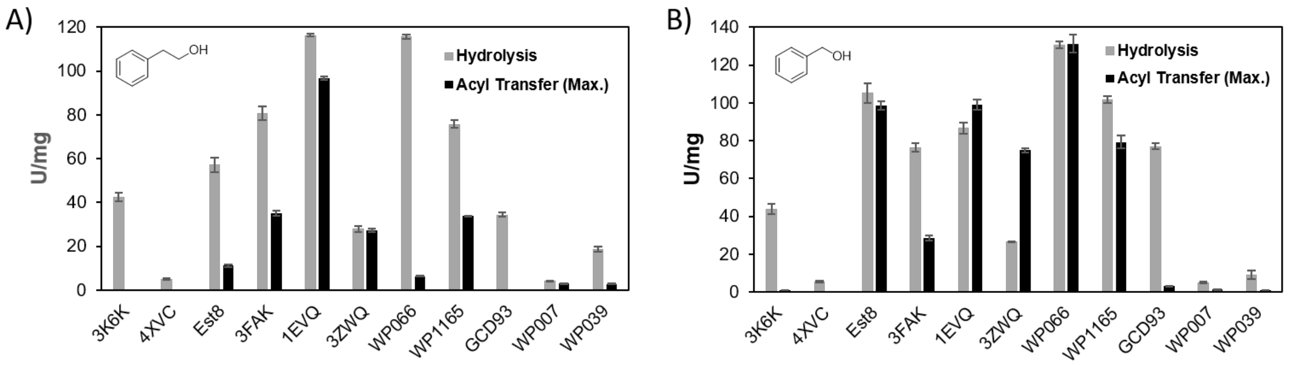

Figure S10: Specific activities for hydrolysis and maximum acyltransfer for all investigated bHSLs in the pNPA assay using 2-phenylethanol (A) and benzyl alcohol (B) as acyl acceptor.

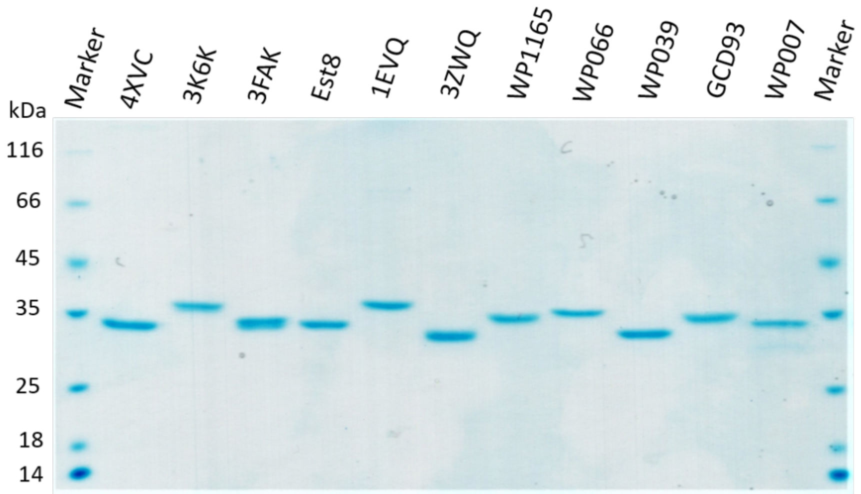

Figure S11: SDS-PAGE analysis of samples of the purified and desalted bHSLs used in this study. MW(3K6K) = 33.09 kDa, MW(4XVC) = 32.09 kDa, MW(Est8) = 31.06 kDa, MW(3FAK) = 31.91 kDa, MW(1EVQ) = 34.30 kDa, MW(3ZWQ) = 34.35 kDa, MW(WP1165) = 33.26 kDa, MW(WP066) = 32.75 kDa, MW(GCD93) = 32.50 kDa, MW(WP039) = 30.89 kDa, MW(WP007) = 31.72 kDa. Pierce™ Unstained Protein MW Marker (Thermofischer) was used.

Sequences

DNA Sequence of 4XVC
ATGGCCAAAAGTCCGGAACTGGATCGTGTGATTGGCATGATTCGCGAACGTGCCGCCACCCCGCGTAAAACCACCGATG
ATGATCGCCGTCTGTATGAAACCATGCTGGGCAGTATGCCGCTGGATGATGATATTCAGACCGAACGCCTGGGTGTTAAT
GGTGTTCCGGCCGAATGGATCTATGCACCGGGTGCCCGTGATGATCAGGTTTTTCTGTATCTGCATGGCGGTGGCTATG
TGATTGGCAGTATGCGCACCCATCGTGTGATGCTGAGTCATATTGCACGCGCCGCCGGCTGTCGCGTGCTGGGTTTAGA
TTATCGTCTGGCACCGGAAACCCCGTTTCCGGCACCGGTTGAAGATACCGTTGCAGCCTATCGCTGGCTGCTGGCACAT
GGCTATGATCCGAGTCGTATTGCACTGGGTGGTGACAGCGCAGGCGGTGGTCTGGTTGTGGCCGCCCTGGTGGCCCTG
CGTTATATTGGTGAACCGCTGCCGGCAGCAGGCGTGTGCCTGTCACCGTGGATTGATATGGAAGCAACCGGCGAAAGTT
TTACCACCAATGCAACAATGGATCCGAGTGTGAATAAGGAACGCGTTATGAGCATTGCAGCCCTGTATCTGGGCGGCAAA
AATCCGCAGGCACCGCTGGCAAGTCCGCTGTATGCCGATCTGCAGGGTCTGCCGCCGCTGCTGGTTCAGGTTGGTGGC
ATTGAAACCCTGCTGGATGATGCCCGCGCCCTGACCACCCGTGCCAAAGCTGCCGGCGTGGATGCCGATCTGGAAGTTT
GGGATGATATGCCGCATGTGTGGCAGCATTTTGCACCGATTCTGCCGGAAGGCAAACAGGCAATTGCACGTATTGGTGA
ATTTCTGCGTAAACAGATTGGCTAA

Amino Acid Sequence of 4XVC
MAKSPELDRVIGMIRERAATPRKTTDDDRRLYETMLGSMPLDDDIQTERLGVNGVPAEWIYAPGARDDQVFLYLHGGGYVIGS
MRTHRVMLSHIARAAGCRVLGLDYRLAPETPFPAPVEDTVAAYRWLLAHGYDPSRIALGGDSAGGGLVVAALVALRYIGEPLP
AAGVCLSPWIDMEATGESFTTNATMDPSVNKERVMSIAALYLGGKNPQAPLASPLYADLQGLPPLLVQVGGIETLLDDARALTT
RAKAAGVDADLEVWDDMPHVWQHFAPILPEGKQAIARIGEFLRKQIG

DNA Sequence of 3K6K
ATGGGCGCAATGGATCAGGAAATTGGCACCGTGACCGATACCAAAATGGATCCGCGTGATTTTCTGCAGCTGCTGAAAAT
TAATGCCGAAAAAGCAGAAAAGAATCTGCCGCTGGATCAGAAACGCGCCGGTATGGAAGCACTGTGTGAACGTTTTCCG
CGCGCCGAAGGCGTGGAACTGACCCTGACCGATCTGGGTGGTGTTCCGTGCATTCGCCAGGCAACCGATGGCGCAGGT
GCAGCCCATATTCTGTATTTTCATGGTGGCGGTTATATTAGCGGTAGTCCGAGTACCCATCTGGTGCTGACCACCCAGCT
GGCAAAACAGAGCAGTGCCACCCTGTGGAGCCTGGATTATCGTCTGGCCCCGGAAAATCCGTTTCCGGCCGCAGTTGAT
GATTGTGTTGCAGCATATCGCGCCCTGCTGAAAACCGCAGGTAGCGCAGATCGTATTATTATTGCCGGTGACAGTGCAG
GCGGTGGTCTGACCACCGCAAGCATGCTGAAAGCAAAAGAAGATGGCCTGCCGATGCCGGCAGGTCTGGTTATGCTGA
GTCCGTTTGTGGATCTGACCCTGAGTCGCTGGAGTAATAGCAATCTGGCCGATCGTGATTTTCTTGCAGAACCGGATACC
CTGGGTGAAATGAGTGAACTGTATGTGGGCGGTGAAGATCGTAAAAATCCGCTGATTAGTCCGGTGTATGCCGATCTGA
GTGGCCTGCCGGAAATGCTGATTCATGTGGGCAGCGAAGAAGCCCTGCTGAGTGATAGTACCACCCTGGCCGAACGTG
CAGGTGCCGCCGGTGTTAGCGTGGAACTGAAAATTTGGCCGGATATGCCGCATGTGTTTCAGATGTATGGCAAATTTGTG
AATGCCGCAGATATTAGCATTAAGGAAATTTGCCATTGGATTAGCGCCCGTATTAGCTAA

Amino Acid Sequence of 3K6K
MGAMDQEIGTVTDTKMDPRDFLQLLKINAEKAEKNLPLDQKRAGMEALCERFPRAEGVELTLTDLGGVPCIRQATDGAGAAHI
LYFHGGGYISGSPSTHLVLTTQLAKQSSATLWSLDYRLAPENPFPAAVDDCVAAYRALLKTAGSADRIIIAGDSAGGGLTTASML
KAKEDGLPMPAGLVMLSPFVDLTLSRWSNSNLADRDFLAEPDTLGEMSELYVGGEDRKNPLISPVYADLSGLPEMLIHVGSEE
ALLSDSTTLAERAGAAGVSVELKIWPDMPHVFQMYGKFVNAADISIKEICHWISARIS

DNA Sequence of 3FAK
ATGGCCGGTCCGGAAATTGTTAAACTGAAAAAGATTCTGCGTGAAAAAGCCGTGCCGCCGGGCACCGAAGTGCCGTTAG
ATGTTATGCGTAAAGGTATGGAAAAAGTGGCATTCAAAGCAGCAGATGATATTCAGGTGGAACAGGTGACCGTTGCAGGT
TGCGCAGCAGAATGGGTTCGCGCACCGGGCTGCCAGGCAGGTAAAGCAATTCTGTATCTGCATGGCGGTGGCTATGTTA
TGGGCAGTATTAATACCCATCGCAGTATGGTGGGTGAAATTAGCCGCGCCAGCCAGGCCGCCGCACTGTTACTGGATTA
TCGTCTGGCCCCGGAACATCCGTTTCCGGCCGCAGTTGAAGATGGTGTTGCAGCATATCGCTGGCTGCTGGATCAGGGT
TTTAAACCGCAGCATCTGAGTATTAGTGGTGACAGCGCAGGCGGTGGTCTGGTTCTGGCCGTGCTGGTTAGTGCACGTG
ATCAGGGTCTGCCGATGCCGGCCAGTGCAATTCCGATTAGCCCGTGGGCAGATATGACCTGTACCAATGATAGCTTTAAA
ACCCGCGCCGAAGCAGATCCGATGGTGGCCCCGGGTGGCATTAATAAGATGGCAGCCCGCTATCTGAATGGTGCCGAT
GCCAAACATCCGTATGCAAGTCCGAATTTTGCAAATCTGAAAGGTCTGCCGCCGCTGCTGATTCATGTTGGTCGTGATGA
AGTTCTGCTGGATGATAGTATTAAGCTGGATGCAAAAGCCAAAGCCGATGGTGTGAAAAGTACCCTGGAAATTTGGGATG
ATATGATTCATGTTTGGCATGCCTTTCATCCGATGCTGCCGGAAGGCAAACAGGCAATTGTGCGTGTGGGCGAATTCATG
CGCGAACAGTGGGCAGCCTAA

Amino Acid Sequence of 3FAK
MAGPEIVKLKKILREKAVPPGTEVPLDVMRKGMEKVAFKAADDIQVEQVTVAGCAAEWVRAPGCQAGKAILYLHGGGYVMGSI
NTHRSMVGEISRASQAAALLLDYRLAPEHPFPAAVEDGVAAYRWLLDQGFKPQHLSISGDSAGGGLVLAVLVSARDQGLPMP
ASAIPISPWADMTCTNDSFKTRAEADPMVAPGGINKMAARYLNGADAKHPYASPNFANLKGLPPLLIHVGRDEVLLDDSIKLDA
KAKADGVKSTLEIWDDMIHVWHAFHPMLPEGKQAIVRVGEFMREQWAA

DNA Sequence of Est8
ATGGCGAGTCCGCAACTACAGATGGCGCTTGATGGGTTCAAGATGATGGGAGAGAAGATGGCGCAGGCCGGTGGGGAC
GTGAAGGCAATGCGTGCCGTTATGGAAGAGATGGCCACCTTTCCCTCGGCAGGAGAAACGAAGTGCACTCCAGTGAATG
CGGGTGGCGTCCCAGCTGAGTGGATTGCTGCTCCGGGGGCAGCGGACGACCGCGTGATCTTGTATCTCCATGGTGGCG
GCTACGTGATGGGCTCTATTACCACGCACCGTGAGACGATCGCACGCTTATCGAAAGCCTCAGGAGCGCGAGCGCTGG
CGCTCGATTATCGCTTAGCTCCGGAGTATCCATTTCCCGCCGCCGTGGATGACGCAACGGCAGCCTATCGCTGGTTGTT
ATCACAAGATATCAAGCCGTCTCGTATTGTCGTGGCTGGAGACTCTGCCGGAGGCGGGCTCGTTCTGGCCACGCTGGTG
GCGCTGCGCGATGCGAAAGTCCCTCTGCCCGCGGCAGGAGTGTGCATTTCACCATGGGCGGATATGGAAGGGACCGGC
GCATCCATGACAACCAGAGCGAAGGCTGATCCGGTGGTGCAAAAAGAGATGCTCGTCAACATGGGAAAGACGTATCTCG
GTGGCAAAGACGCAAAATCACCGCTCGCGGCTCCACTTCATGCTGATTTCCGAGGACTGCCCCCGCTGTTCATTCAGGT
TGGCGACGCCGAGACGTTGCTTGATGACTCCACCCGTGTTGCGGAAAAGGCGAAGATGGCTGGGGTCAAGGTGGATCT
CGAGATCTGGCCGGAGATGCCACACGTATGGCATCTATTTGCTCCTTTCCTACCGGAAGGGCAACAAGCCATCGATAAG
ATCGGCCAGTACGTAAAGCAGCGAACTGCTTAG

Amino Acid Sequence of Est8
MASPQLQMALDGFKMMGEKMAQAGGDVKAMRAVMEEMATFPSAGETKCTPVNAGGVPAEWIAAPGAADDRVILYLHGGGY
VMGSITTHRETIARLSKASGARALALDYRLAPEYPFPAAVDDATAAYRWLLSQDIKPSRIVVAGDSAGGGLVLATLVALRDAKVP
LPAAGVCISPWADMEGTGASMTTRAKADPVVQKEMLVNMGKTYLGGKDAKSPLAAPLHADFRGLPPLFIQVGDAETLLDDST
RVAEKAKMAGVKVDLEIWPEMPHVWHLFAPFLPEGQQAIDKIGQYVKQRTA

DNA Sequence of 1EVQ
ATGCCGCTGGATCCGGTTATTCAGCAGGTGCTGGATCAGCTGAATCGTATGCCGGCCCCGGATTATAAACATCTGAGTG
CACAGCAGTTTCGTAGTCAGCAGAGCCTGTTTCCGCCGGTGAAAAAAGAACCGGTGGCAGAAGTTCGTGAATTTGATATG
GATCTGCCGGGTCGTACCCTGAAAGTTCGTATGTATCGTCCGGAAGGTGTGGAACCGCCGTATCCGGCCCTGGTTTATT
ATCATGGCGGTGGTTGGGTTGTGGGCGATCTGGAAACCCATGATCCGGTGTGCCGCGTTCTGGCCAAAGATGGTCGCG
CCGTGGTGTTTAGCGTTGATTATCGTCTGGCACCGGAACATAAATTTCCGGCCGCCGTTGAAGATGCATATGATGCACTG
CAGTGGATTGCAGAACGTGCCGCAGATTTTCATCTGGATCCGGCCCGCATTGCAGTTGGTGGTGACAGCGCCGGTGGTA
ATCTGGCAGCAGTGACCAGTATTCTGGCAAAAGAACGCGGCGGCCCGGCACTGGCATTTCAGCTGCTGATCTATCCGAG
CACCGGTTATGATCCGGCACATCCGCCGGCAAGTATTGAAGAAAATGCCGAAGGTTATCTGCTGACCGGTGGTATGATG
CTGTGGTTTCGCGATCAGTATCTGAATAGCCTGGAAGAACTGACCCATCCGTGGTTTAGTCCGGTTCTGTATCCGGATCT
GAGCGGCCTGCCGCCGGCCTATATTGCCACCGCACAGTATGATCCGCTGCGCGATGTGGGTAAACTGTATGCAGAAGC
ACTGAATAAGGCAGGTGTGAAAGTGGAAATTGAAAATTTTGAAGACCTGATTCACGGTTTTGCCCAGTTTTATAGCCTGAG
CCCGGGCGCCACCAAAGCACTGGTTCGCATTGCCGAAAAACTGCGCGATGCACTGGCC

Amino Acid Sequence of 1EVQ
MPLDPVIQQVLDQLNRMPAPDYKHLSAQQFRSQQSLFPPVKKEPVAEVREFDMDLPGRTLKVRMYRPEGVEPPYPALVYYHG
GGWVVGDLETHDPVCRVLAKDGRAVVFSVDYRLAPEHKFPAAVEDAYDALQWIAERAADFHLDPARIAVGGDSAGGNLAAVT
SILAKERGGPALAFQLLIYPSTGYDPAHPPASIEENAEGYLLTGGMMLWFRDQYLNSLEELTHPWFSPVLYPDLSGLPPAYIATA
QYDPLRDVGKLYAEALNKAGVKVEIENFEDLIHGFAQFYSLSPGATKALVRIAEKLRDALA

DNA Sequence of 3ZWQ
ATGCCTCTTAGCCCTATACTAAGGCAAATTCTCCAACAGTTGGCCGCGCAGTTGCAGTTTAGACCCGACATGGACGTCAA
GACGGTGAGAGAGCAGTTTGAGAAGTCCTCCCTCATCCTCGTCAAAATGGCCAATGAGCCTATTCACCGTGTGGAGGAC
ATCACGATTCCGGGCAGGGGCGGGCCAATTAGGGCTAGGGTTTATAGGCCGCGGGATGGGGAGAGGTTGCCCGCGGT
GGTGTACTACCACGGCGGGGGCTTCGTCTTGGGGAGCGTGGAGACTCACGACCACGTGTGTAGGCGGTTGGCCAACCT
CTCCGGGGCAGTCGTCGTCTCTGTGGACTACCGCCTAGCCCCCGAGCACAAATTCCCCGCCGCCGTGGAAGACGCATA
CGACGCCGCCAAGTGGGTCGCCGACAACTACGACAAGCTCGGCGTCGACAATGGGAAAATCGCCGTGGCTGGGGACTC
GGCGGGGGGCAACTTAGCCGCGGTGACGGCCATCATGGCCAGGGACAGGGGGGAGAGCTTTGTGAAATACCAAGTGTT
AATCTACCCCGCGGTCAACCTCACTGGGTCTCCCACAGTGTCTAGAGTGGAGTACAGCGGGCCCGAATACGTCATCCTC
ACCGCCGACTTAATGGCGTGGTTTGGGAGACAGTATTTCTCAAAGCCGCAAGACGCCCTCAGTCCCTATGCCTCTCCCAT
ATTTGCAGATTTGTCAAACCTCCCGCCCGCCCTGGTGATAACCGCCGAGTACGACCCGCTACGCGACGAGGGAGAGCTC
TACGCCCACTTGTTGAAGACTAGGGGAGTTAGGGCCGTGGCGGTGAGGTACAACGGCGTCATCCACGGCTTCGTCAACT
TCTACCCCATATTAGAAGAGGGGAGAGAGGCAGTTTCGCAAATTGCGGCCTCAATAAAGTCGATGGCTGTGGCGTAA

Amino Acid Sequence of 3ZWQ

MPLSPILRQILQQLAAQLQFRPDMDVKTVREQFEKSSLILVKMANEPIHRVEDITIPGRGGPIRARVYRPRDGERLPAVVYYHGG
GFVLGSVETHDHVCRRLANLSGAVVVSVDYRLAPEHKFPAAVEDAYDAAKWVADNYDKLGVDNGKIAVAGDSAGGNLAAVTA
IMARDRGESFVKYQVLIYPAVNLTGSPTVSRVEYSGPEYVILTADLMAWFGRQYFSKPQDALSPYASPIFADLSNLPPALVITAE
YDPLRDEGELYAHLLKTRGVRAVAVRYNGVIHGFVNFYPILEEGREAVSQIAASIKSMAVA

DNA Sequence of WP_116581250.1

ATGCCGCTGGATAAACAGATTGCCGGTGTGCTGCAGCAGTTTCGCGATCTGCGTGAACCGGATTTTAGTCAGGTGGATG
CCGCACAGTATCGTCAGTTTAGTGATAATCTGCTGCCGGCAATTCCGGGTGACCCGATGAGCGAAGTTCGCGATCTGAA
AGTTGCCGGCGCAGATGGTGACCTGGATGCCCGTCTGTATCGTCCGAGCGAAGCCCCGAATCTGCCGCTGCTGGTTTTC
TTTCATGGCGGCGGTTTTGTTATGGGCAATCTGGATACCCATGATAATCTGTGTCGCAGTCTGGCCCGCCAGACCGAAG
CAGTTGTGGTGAGTGTTGCATATCGTCTGGCACCGGAACATAAATTTCCGGTGGCACCGCTGGATTGTTATGCCGCAACC
TGTTGGCTGGTGGCCCATGCAGCCGAACTGGGCTTTGATGGCAGCCGCCTGGCAGTGGCCGGTGACTCAGCCGGTGG
CAATCTGGCCCTGGCAGTTAGTCGTCTGGCAGCCCAGGGTAAAGGCCCGAAAATTAGTTATCAGTGCCTGTTTTATCCGG
TTACCGATGCAGGTTGCGATAGTCAGAGTTTTGAAGAATTTGCCGAAAGTTATCTGCTGAGTGCCAAAGCCATGCGTTGG
TTTTGGCAGCAGTATCTGCAGGAAGATGGCCAGGCCGATGATCCGCTGGCAAGTCCGCTGCGTGCCGAAAGTCTGGCA
GGTCTGCCGCCGACCACCCTGTTTACCGCAGCATTTGATCCGCTGCGCGATGAAGGCGAAGCCCTGGCAGAATGTCTG
CGCGAAGCCGGCGTGGCAGTTCTGGTGCAGCGCTATGAAGGTATGATTCATGGCTTTATTAGTATGGCCCCGTTTGTTG
AAGCCGCCGCACAGGCCCTGACCGATGCCTGTGCCGATCTGCGCGGTGCACTGCAG

Amino Acid Sequence of WP_116581250.1

MPLDKQIAGVLQQFRDLREPDFSQVDAAQYRQFSDNLLPAIPGDPMSEVRDLKVAGADGDLDARLYRPSEAPNLPLLVFFHG
GGFVMGNLDTHDNLCRSLARQTEAVVVSVAYRLAPEHKFPVAPLDCYAATCWLVAHAAELGFDGSRLAVAGDSAGGNLALAV
SRLAAQGKGPKISYQCLFYPVTDAGCDSQSFEEFAESYLLSAKAMRWFWQQYLQEDGQADDPLASPLRAESLAGLPPTTLFT
AAFDPLRDEGEALAECLREAGVAVLVQRYEGMIHGFISMAPFVEAAAQALTDACADLRGALQ

DNA Sequence of WP_066869841.1

ATGCGCCTGGATCCGCAGATTGCACCGATTATTGAACAGCTGGATAGCGGCTTTCCGCCGGTTCATCAGATGAGTGGCG
CAGAAGCCCGCGCCCTGATTCGTAGTCGCCTGGTTCCGCCGGCCCGTCCTGAACCTGTTGCAGAAGTTACCGATCGCA
GTATTGAAGGTCAGGGTGGTCCGATTCCGGTGCGTAGTTATCGTCCGGAAGCAGCAGGTCCGCTGCCGGTGGTGGTGT
ATGCACATGGCGGCGGTTTTGTGTTTTGCGATCTGGATAGCCATGATGATCTGTGTCGTAGCCTGGCCAATCTGGTTCCG
GCCGTGGTGGTGAGTGTGGGTTATCGCCTGGCACCGGAAAATACCTGGCCGGCCGCAGCAGAAGATGTGTATGCAGCC
ACCTGCTGGGCCTATGATAATGCAGCCGCCCTGGGCAGCGATCCGGGTAGACTGGTTGTGGGTGGTGACAGTGCAGGT
GGTAATCTGGCCGCAGTGGCAACCGTGATTAGTCGCGATCGTGGCGGTCCGATGCCGGCCGCACAGTTACTGATCTATC
CGGTTATTGCAGCAGATTTTGATACCGAAAGCTATCGCCTGTTTGGTCAGGGTTATTATAATCCGAAACCGGCACTGCGT
TGGTATTGGGATTGTTATGTGCCGAGTAGCGAAGATCGTGCCCATCCGTATGCCACCCCGCTGAATGCCGATCTGCGCG
GTCTGCCGCCGGCCGTGTTAGTGGTGGCAGGCCATGATCCGCTGCGCGATGAAGGTCTGGCCTTTGGTGCCGCACTGG
AAGCCGCCGGCGTGCCTACACGCCAGCTGAGATATGAAGGCGGTATTCATGGTTTTATGACCATGCCGATGCTGGATCT
GGCCCATCGCGCCCGCAATGAAGCAGCCGCCGCACTGGCCGATCTGCTGCGTCGT

Amino Acid Sequence of WP_066869841.1

MRLDPQIAPIIEQLDSGFPPVHQMSGAEARALIRSRLVPPARPEPVAEVTDRSIEGQGGPIPVRSYRPEAAGPLPVVVYAHGGG
FVFCDLDSHDDLCRSLANLVPAVVVSVGYRLAPENTWPAAAEDVYAATCWAYDNAAALGSDPGRLVVGGDSAGGNLAAVAT
VISRDRGGPMPAAQLLIYPVIAADFDTESYRLFGQGYYNPKPALRWYWDCYVPSSEDRAHPYATPLNADLRGLPPAVLVVAGH
DPLRDEGLAFGAALEAAGVPTRQLRYEGGIHGFMTMPMLDLAHRARNEAAAALADLLRR

DNA Sequence of GCD93300.1

ATGACCATTGATCCGCAGCTGGTGGAACTGCTGGGTGTTCTGCCGGATGATCTGTTCGTTGGCGATGCAGCAACCGCCC
GTGCCACCATTGATGCAGGTGTTGCACTGCTGCCGGTTCTGACCGAACTGGCACGCGTGGAAGATCGCACCGTTCCGG
GCGCCGTGGATGCACGTCCGGCAAGAGTGTATCGCCCGAGTGAAGAACCGGGTCTGCCGGTTACCCTGTTCTTCCATG
GTGGCGGCTTCGTTATTGGTGGCCTGGATAGCCATGATGCCCTGGCACGCCGTATTGCACGCCATGCAAATTGCGTGGT
TGTGAGCACCGATTATCGTCTGGCCCCGGAATTCCCGTTCCCGGCAGCAACCGAAGATGCATTCGCAGCATATCGCTGG
CTGCTGGATCATGCAGGCGAACTGGGCGCAGATCCGACCCGCATTGCACTGGCAGGCGATAGTGCCGGCGCAACCCTG
AGCGCAACCGTGTGTCTGCTGGCACGTGAACATGGTCTGCCGCAGCCGGCCCTGCAGATGCTGTGGTATCCGACCACC
GGCGTGGAAGGCACCGCCAGTCAGGTTGAAAATGCAGATGCACCGCTGCTGACCGCAGCAAGTATTGCATGGTTCCTG
GGCCATTACTTCGGCGGCCGTGATCTGGATGATCTGGGTCCGTATGCACGCATTGGTACCATTGAAGATCTGAGCGGCC
TGGCCCCGGCCCATGTGGTTATTGCCGGTCATGATCCGCTGCGCGATGAAGGTGCAGATTATGCAGAACGTCTGCGCG
CCGCCGGCAATGCCGTTGAATGCGAAAGCTTCGATGATATGGTGCATGGCTTCATGAGCTTCGTTGATCTGGTGCCGCG
CTGTGAAGAATGCGCCACCGGTAGCTTCACCGCCCTGCGCCGTGCCCTGCATCCG

Amino Acid Sequence of GCD93300.1
MTIDPQLVELLGVLPDDLFVGDAATARATIDAGVALLPVLTELARVEDRTVPGAVDARPARVYRPSEEPGLPVTLFFHGGGFVI
GGLDSHDALARRIARHANCVVVSTDYRLAPEFPFPAATEDAFAAYRWLLDHAGELGADPTRIALAGDSAGATLSATVCLLARE
HGLPQPALQMLWYPTTGVEGTASQVENADAPLLTAASIAWFLGHYFGGRDLDDLGPYARIGTIEDLSGLAPAHVVIAGHDPLR
DEGADYAERLRAAGNAVECESFDDMVHGFMSFVDLVPRCEECATGSFTALRRALHP

DNA Sequence of WP_007531565.1
CCATGCCGTATCTGCCGCTGCCGGTTGTTGCCGGTGCCCTGGCTCCGTTCTATCGTCTGGCACTGCATCCGGCCCTGCC
GGCACCTGTGGCACGTAGAATTATTGATGCCGGTAGTGTTCTGCAGGCACTGCCGCGTGATACCGTTGTTCGTCCGCTG
ACCCTGGCCGGTCGCCCTGCAGAACGTATTACCGTGGGTGCCACCGAACGCCGCACCGCTATTCTGTATCTGCATGGTG
GCGGCTATACCCTGGGTAGCCTGGATACCCATCGCAGCCTGGCAGCCCATCTGGCACGTGAAAGTGGCAGTGCAGTGT
ATGTTCTGGATTATCGCCTGGCACCGGAACATCCGTATCCGGCAGCAGTGGAAGATGCCGTTGCAGCATATCTGGAACT
GCTGGGCGAACATGGTCTGACCCCGGAAACCTTAGCCGTGGCCGGCGATAGCGCAGGCGGCGGTCTTAGTCTGGCAAC
CGCCCGTCGTCTGGTTGATCGTTATAGTGTGAAACCGGCAGCACTGGCACTGATTGCACCGTGGGTGGATCCGGGCGC
ACGTGATGCCCCGTTCGATCGTGATACCGTGATTAATACCGGTTGGAGCCATCGTGCCGCCGCAGCATATCTTGGTGAT
GGCGATGCACGCGATCCGGGTTATGCCCCGCTGCTGGGTGATCTGAGTGGCCTGCCGCCGACCGTTGTGCATGTGGGC
ATGAGTGAAGTTCTGTATCCGCAGGTTGTTGATCTGGTGGATCGCATGCGTAGTGCAGGCACCACCGTGGCATATACCG
AACATCCGACCCTGTGGCATGTGGCCCATCTGCAGGCAAGTCTGGTGCGCGAAAGTGCCGATGCAGTGGCCGAACTGG
GTGAATTCCTGCGTGCACGTCTGAGTGCACAGCCGGCCTAACTCGAG

Amino Acid Sequence of WP_007531565.1
MPYLPLPVVAGALAPFYRLALHPALPAPVARRIIDAGSVLQALPRDTVVRPLTLAGRPAERITVGATERRTAILYLHGGGYTLGSL
DTHRSLAAHLARESGSAVYVLDYRLAPEHPYPAAVEDAVAAYLELLGEHGLTPETLAVAGDSAGGGLSLATARRLVDRYSVKP
AALALIAPWVDPGARDAPFDRDTVINTGWSHRAAAAYLGDGDARDPGYAPLLGDLSGLPPTVVHVGMSEVLYPQVVDLVDRM
RSAGTTVAYTEHPTLWHVAHLQASLVRESADAVAELGEFLRARLSAQPA

DNA Sequence of WP_039884751.1
ATGAGTCTGGTGGAACTGGATGCCCTGCTGGCAGTGATGGCCGCCAATCCGCCGCCGCAGGGTGGTCCTCTGGAACTG
CGTGATTGGTTCAATGTTGCCCATGCCGCACTGCCGGTTCCGGAAGGCCTGGAAATTCGTCGCGTGGAAGCCGGTCCG
GTTGGCGGTGATCTGATTCTGCCGGCCGATGCAGTTCCGGGTCGTCTGATTATCTATTATCATGGCGGTGGCTTCGTTCT
GGGTAGCGCCCGCACCCATCGCACCGTTGCAGCAAATCTGGCCCGCGCAGCAAATATTGCAGTGCTGGCAGCCGATTA
TCGCCTGGCACCGGAACATGCCTTCCCGGCCGCCCATGATGATGCACTGAGTGCCTTCCATTGGGCACTGGCAGAAGG
CTATGAAGCAATTGCACTGAGCGGTGATAGCGCCGGTGGCAATCTGGCCCTGAGCACCGCCGTGCGCGCCCGTAATGG
CGGTGGTCAGAGCCCGGCCGCCCTGGCATTAATGAGTCCGGCCCTGGACTTCGCCGGTGATGGCGGTACCCATCATAG
CGTTAGCGATGATCCGATTCTGAGCAAAGAACTGGTGGATCTGTTCCTGAGTGCCTATCTGCCGGGTCAGAGCCTGCGT
GATCCGGCAGTGACCCCGCTGTTCGCCGAACTGAGTGGTCTGCCGCCGGTTCTGGTGCATGTGGGCAGCCGCGAAATG
CTGCGCGATGATAGCGTGACCATTGCACGTCGCCTGCGTGATGCCGGTGTTCAGGCAGAACTGCGTGTGTGGGATGGC
ATGTGCCATAGCTGGCAGCTGTATGCCCCGCTGGTGCGTGAAGGTCTGGATAGCATTGAAGAAGTGGCCAGCTTCCTGC
GCGGTCATCTGGTGGCAAGC

Amino Acid Sequence of WP_039884751.1
MSLVELDALLAVMAANPPPQGGPLELRDWFNVAHAALPVPEGLEIRRVEAGPVGGDLILPADAVPGRLIIYYHGGGFVLGSART
HRTVAANLARAANIAVLAADYRLAPEHAFPAAHDDALSAFHWALAEGYEAIALSGDSAGGNLALSTAVRARNGGGQSPAALAL
MSPALDFAGDGGTHHSVSDDPILSKELVDLFLSAYLPGQSLRDPAVTPLFAELSGLPPVLVHVGSREMLRDDSVTIARRLRDAG
VQAELRVWDGMCHSWQLYAPLVREGLDSIEEVASFLRGHLVAS

References

[1] *The Proteomics Protocols Handbook, Humana Press, Totowa, N.J.,* ***2005***.
[2] U. K. Laemmli, *Nature* **1970**, *227*, 680-685.
[3] K. M. Sparta, M. Krug, U. Heinemann, U. Mueller, M. S. Weiss, *J. Appl. Crystallogr.* **2016**, *49*, 1085–1092.
[4] A. J. McCoy, R. W. Grosse-Kunstleve, P. D. Adams, M. D. Winn, L. C. Storoni, R. J. Read, *J. Appl. Crystallogr.* **2007**, *40*, 658-674.
[5] K. Diederichs, P. A. Karplus, *Nat. Struct. Biol.* **1997**, *4*, 269–275.
[6] M. S. Weiss, R. Hilgenfeld, *J. Appl. Crystallogr.* **1997**, *30*, 203–205.
[7] P. A. Karplus, K. Diederichs, *Science* **2012**, *336*, 1030–1033.
[8] D. J. Abraham, A. J. Leo, *Proteins* **1987**, *2*, 130-152.
[9] a) T. Halgren, *Chem. Biol. Drug Des.* **2007**, *69*, 146-148; b) T. A. Halgren, *J. Chem. Inf. Model.* **2009**, *49*, 377-389.
[10] P.-Y. Li, X.-L. Chen, P. Ji, C.-Y. Li, P. Wang, Y. Zhang, B.-B. Xie, Q.-L. Qin, H.-N. Su, B.-C. Zhou et al., *J. Biol. Chem.* **2015**, *290*, 11188-11198.
[11] K. H. Nam, M.-Y. Kim, S.-J. Kim, A. Priyadarshi, S.-T. Kwon, B.-S. Koo, S.-H. Yoon, K. Y. Hwang, *Proteins* **2009**, *74*, 1036-1040.
[12] K. H. Nam, M.-Y. Kim, S.-J. Kim, A. Priyadarshi, W. H. Lee, K. Y. Hwang, *Biochem. Biophys. Res. Commun.* **2009**, *379*, 553-556.
[13] G. Manco, E. Giosuè, S. D'Auria, P. Herman, G. Carrea, M. Rossi, *Arch. Biochem. Biophys.* **2000**, *373*, 182-192.
[14] Y. Hotta, S. Ezaki, H. Atomi, T. Imanaka, *Appl. Environ. Microbiol.* **2002**, *68*, 3925-3931.
[15] L. Mestrom, J. G. R. Claessen, U. Hanefeld, *ChemCatChem* **2019**, *11*, 2004-2010.

Author Contributions

U.T.B and H.M. conceived the project. H.M. designed and performed most experiments. A.K.B. processed the sequence library and colorimetric assay data. L.B. purified and crystallyzed the enzyme. G.J.P and L.B. performed the crystallographic data collection and processing. U.T.B. directed the project. H.M. drafted the manuscript to which all authors contributed.
